# Balanced Free Essential Amino Acids and Resistance Exercise Training Synergistically Improve Dexamethasone-Induced Impairments in Muscle Strength, Endurance, and Insulin Sensitivity in Mice

**DOI:** 10.3390/ijms23179735

**Published:** 2022-08-27

**Authors:** Jiwoong Jang, Jin-Ho Koh, Yeongmin Kim, Hee-Joo Kim, Sanghee Park, Yewon Chang, Jiyeon Jung, Robert R. Wolfe, Il-Young Kim

**Affiliations:** 1 Korea Mouse Metabolic Phenotyping Center, Lee Gil Ya Cancer and Diabetes Institute, Gachon University, Incheon 21999, Korea; 2 Department of Internal Medicine, Gil Medical Center, Gachon University, Incheon 21565, Korea; 3 Department of Molecular Medicine, College of Medicine, Gachon University, Incheon 21565, Korea; 4 Department of Health Sciences and Technology, GAIHST, Gachon University, Incheon 21999, Korea; 5 Department of Geriatrics, Center for Translational Research in Aging and Longevity, Donald W. Reynolds Institute on Aging, University of Arkansas for Medical Sciences, Little Rock, AR 72205, USA

**Keywords:** dexamethasone, muscle atrophy, essential amino acids, resistance exercise training, physical performance, mitochondrial biogenesis, neuromuscular junction stability, protein turnover, glucose metabolic flux

## Abstract

Our previous study shows that an essential amino acid (EAA)-enriched diet attenuates dexamethasone (DEX)-induced declines in muscle mass and strength, as well as insulin sensitivity, but does not affect endurance. In the present study, we hypothesized that the beneficial effects will be synergized by adding resistance exercise training (RET) to EAA, and diet-free EAA would improve endurance. To test hypotheses, mice were randomized into the following four groups: control, EAA, RET, and EAA+RET. All mice except the control were subjected to DEX treatment. We evaluated the cumulative rate of myofibrillar protein synthesis (MPS) using ^2^H_2_O labeling and mass spectrometry. Neuromuscular junction (NMJ) stability, mitochondrial contents, and molecular signaling were demonstrated in skeletal muscle. Insulin sensitivity and glucose metabolism using ^13^C_6_-glucose tracing during oral glucose tolerance tests were analyzed. We found that EAA and RET synergistically improve muscle mass and/or strength, and endurance capacity, as well as insulin sensitivity, and glucose metabolism in DEX-treated muscle. These improvements are accomplished, in part, through improvements in myofibrillar protein synthesis, NMJ, fiber type preservation, and/or mitochondrial biogenesis. In conclusion, free EAA supplementation, particularly when combined with RET, can serve as an effective means that counteracts the adverse effects on muscle of DEX that are found frequently in clinical settings.

## 1. Introduction

Dexamethasone (DEX), one of the synthetic corticosteroids, is used extensively in the medical field to treat a variety of inflammatory diseases, such as multiple sclerosis, allergies, asthma, atopic dermatitis, contact dermatitis [1], and neuromuscular disorders [2]. However, the chronic use of DEX causes severe side effects, such as impaired proteostasis and glucose metabolism, leading to muscle-wasting and a decrease in physical function, including muscle strength and endurance capacity [3,4].

Glucocorticoid treatment induces a decrease in myoblast determinant protein 1 (MyoD) expression in myotubes via the glucocorticoid receptor (GR), thereby inhibiting myogenesis [5] and reducing the rate of myofibrillar protein synthesis (MPS) [4]. Moreover, DEX treatment causes impairments in protein turnover (i.e., protein breakdown exceeding synthesis) via the induction of the ubiquitin–proteasome system (UPS) and autophagy activity, as well as a decrease in myogenesis [3]. Prolonged DEX treatment decreases not only muscle mass, but also mitochondrial content and oxidative capacity in muscle [6], thereby leading to decreases in muscle strength and endurance [7]. Therefore, it is desirable to discover therapeutic means that can offset the adverse side effects (e.g., deterioration in muscle mass and function and glucose metabolism) while preserving the therapeutic benefits of DEX. However, no previous studies that attempted to discover an effective means were performed with a comprehensive evaluation of protein turnover, body function, and metabolism [3,8,9].

While resistance exercise training (RET) is a well-known, potent anabolic stimulus for improving muscle mass and strength, RET does not induce significant gains in muscle mass without the provision of proper nutrition [10]. For example, it is shown that the supplementation of protein or EAA during RET shows a more adaptational gain in muscle mass and physical function than RET alone [11]. Furthermore, it is EAA, not non-essential amino acids (NEAAs) that induce the stimulation of MPS following the consumption of protein, free amino acids, or food containing them [12]. Furthermore, it is typically well-appreciated that RET does not improve aerobic capacity (i.e., endurance), due partly to a nil effect on mitochondrial biogenesis in skeletal muscle [13]. Although a combination of RET and aerobic exercise training (AET) could be a potential means, it is also well-known that AET counteracts the hypertrophying effect of RET via the inhibition of a key signaling pathway (AMPK–mTORC1 signaling pathway) responsible for stimulation of MPS [14,15]. Therefore, it is ideal if an alternative means exists that alleviates declines in both muscle mass and strength, as well as endurance, due to DEX treatment. In this regard, the addition of free EAA supplementation to RET may fulfill these requirements: EAA play key roles as stimuli and also as precursors for MPS [16], which may include not only contractile proteins but also mitochondrial proteins and, thus, endurance [17]. For example, it is shown that free EAA supplementation for 3 months improves endurance capacity largely through the stimulation of mitochondrial biogenesis in both sedentary and AET mice [17]. We also found in our previous study that EAA, when combined with RET, increases the gene expression of the MuSK, which is known to contribute to neuromuscular junction (NMJ) stability, the effect being linearly associated with muscle strength [11]. Therefore, these findings suggest that EAA supplementation could be an effective strategy that fulfills both aspects: improvements in muscle strength and endurance, as well as insulin sensitivity.

Here, we demonstrate that EAA supplementation and RET synergistically protect muscle from the DEX-induced side effects, at least in part through positively affecting the rate of MPS, NMJ stability, preservation of muscle fiber types, and mitochondrial biogenesis.

## 2. Results

### 2.1. EAA Supplementation and RET Synergistically Prevent DEX-Induced Declines in Muscle Mass and Physical Function

**Figure 1:**
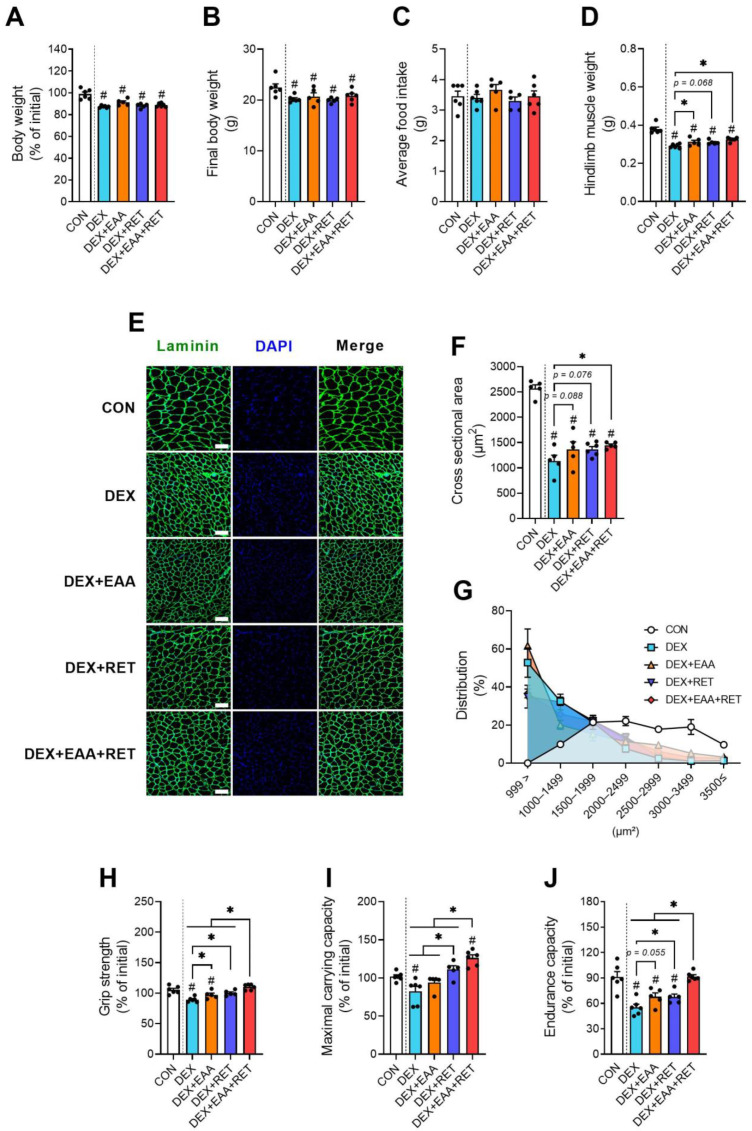
EAA and RET synergistically improve DEX-induced declines in muscle strength and endurance. (**A**) The changes in body weight after 14 days of each treatment (n = 5–6). (**B**) The final body weight after 14 days of each treatment (n = 5–6). (**C**) Average food intake over 14 days (n = 5–6). (**D**) Total hindlimb muscle mass (soleus, plantaris, gastrocnemius, extensor digitorum longus, and tibialis anterior) (n = 5–6). (**E**) Representative image of a gastrocnemius muscle cross-section with laminin (green) and DAPI (blue) staining. Scale bar, 100 μm. (**F**) Cross-sectional area in gastrocnemius. (**G**) Myofiber size frequency distribution of gastrocnemius (n = 5–6). (**H**) Changes in four-limb grip strength over 14 days (n = 5–6). (**I**) Changes in maximal carrying capacity over 14 days (n = 5–6). (**J**) Changes in endurance capacity over 14 days (n = 5–6). Data are presented as mean ± S.E. # Significant difference from control group (*p* < 0.05). * Significant difference between labeled groups (*p* < 0.05). CON, sedentary control; DEX, dexamethasone; EAA, essential amino acids; RET, resistance exercise training.

Following respective treatments, we found that DEX treatment significantly reduces body weight in all groups compared to the control group, regardless of EAA and RET intervention (Figure 1A,B). DEX, EAA, and RET do not affect food consumption (Figure 1B). However, while DEX substantially decreases the hindlimb muscle mass and muscle fiber cross-sectional area compared to control, EAA supplementation and/or RET partially prevents DEX-induced muscle atrophy (Figure 1C–E) and shifting towards smaller muscle fiber size (Figure 1F). In addition, it is well-known that DEX also attenuates muscle strength and endurance capacity [3,4]. Grip strength, maximal carrying capacity (MCC, muscle strength), and treadmill exhaustion tests (endurance capacity) show that RET and EAA supplementation after resistance exercise (EAA+RET) for 14 days not only completely blocks a loss in muscle strength induced by DEX, but also increases the MCC, more than that of the control group (Figure 1G,H). While all treatments improve muscle mass against DEX treatment, the improvement in impaired muscle strength are largely different among the interventions, implying alterations in muscle quality (muscle strength normalized to muscle mass). Interestingly, while EAA or RET alone partially prevents the DEX-induced decline in endurance capacity, the combined treatment completely blocks it (Figure 1I).

Taken together, these results suggest that free EAA supplementation, particularly when combined with RET, can effectively prevent the DEX-induced declines in muscle mass and strength, as well as endurance. Interestingly, endurance capacity is not restored when EAA are fed to mice in a mixed diet (i.e., EEA-enriched diet) [4], indicating that EAA consumption is an important determinant in effectively improving endurance. 

### 2.2. EAA and/or RET Stimulate Muscle Protein Synthesis Rate and Suppression of Autophagy Activation in Muscle of DEX-Treated Mice

**Figure 2:**
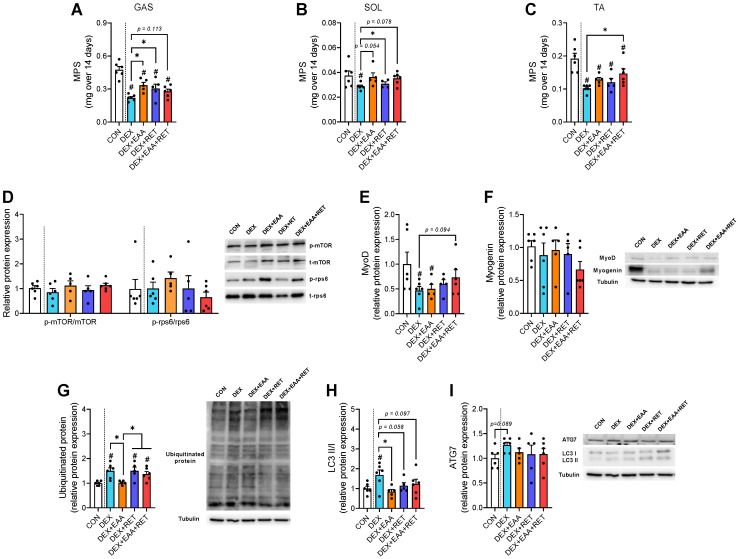
EAA and/or RET stimulate muscle protein synthesis rate and suppression of autophagy activation in muscle of DEX-treated mice. Integrated myofibrillar protein synthesis rate in (**A**) gastrocnemius muscle (mixed muscle), (**B**) soleus muscle (slow oxidative muscle), and (**C**) tibialis anterior muscle (fast glycolytic muscle) for 14 days (n = 5–6 per muscle type). (**D**) Representative image and relative protein expression of mTOR and rps6 in gastrocnemius (n = 5–6). Representative image and relative protein expression of (**E**) MyoD and (**F**) myogenin in gastrocnemius (n = 5–6). (**G**) Representative image and relative protein expression of ubiquitinated-protein in gastrocnemius (n = 5–6). (**H**) Representative image and relative protein expression of the ratio of LC3B II/I and (I) ATG7 in gastrocnemius (n = 5–6). Data are presented as mean ± S.E. # Significant difference from control group (*p* < 0.05). * Significant difference between labeled groups (*p* < 0.05). CON, sedentary control; DEX, dexamethasone; EAA, essential amino acids; RET, resistance exercise training; MPS, myofibrillar protein synthesis; mTORC1, mammalian target of rapamycin complex 1; rps6, ribosomal protein s6; MyoD, myoblast determination protein 1; LC3, microtubule-association protein 1A/1B-light chain 3; ATG7, autophagy-related 7; GAS, gastrocnemius muscle; SOL, soleus muscle; TA, tibialis anterior muscle.

Muscle mass, the main determinant of muscle strength [18], is regulated by the balance between rates of muscle protein synthesis and breakdown [19], an imbalance of which can lead to a loss in muscle mass, as in the case of DEX treatment. To elucidate muscle protein kinetic mechanisms by which each intervention restores the DEX-induced decline in muscle mass, we performed a ^2^H_2_O incorporation study and found that EAA and RET attenuate DEX-induced declines in muscle protein synthesis rates in mixed (gastrocnemius), oxidative (soleus), and/or fast-glycolytic (tibialis anterior) muscle fibers (Figure 2A–C). We found no corresponding changes in implicated singling pathways for MPS, such as mTORC1 activity and myogenin abundance among groups (Figure 2D,F). However, MyoD abundance is reduced in DEX, which recovers, to some extent, with the combined treatment (Figure 2E). The ubiquitin–proteasome system (UPS) and autophagy are involved in muscle atrophy conditions, such as occurring due to DEX [20,21]. We found that DEX increases the expression of ubiquitinated protein and the ratio of LC3B-II/I, an autophagosome marker, which is blocked by EAA (Figure 2G,H). ATG7, which plays a central role in autophagosome biogenesis, tends to increase with DEX, whereas EAA and RET do not change the protein abundance. Taken together, our findings indicate that MPS is stimulated by EAA or RET alone while myofibrillar protein breakdown (MPB), reflected by the activation of UPS or autophagy, is largely suppressed by EAA, which might lead to robust improvements in mass and function (i.e., strength and endurance) of the DEX-treated muscles with the combined treatment.

### 2.3. Combined Treatment of EAA and RET Restores a Decrease in NMJ Stability in Skeletal Muscle Due to DEX Treatment

**Figure 3:**
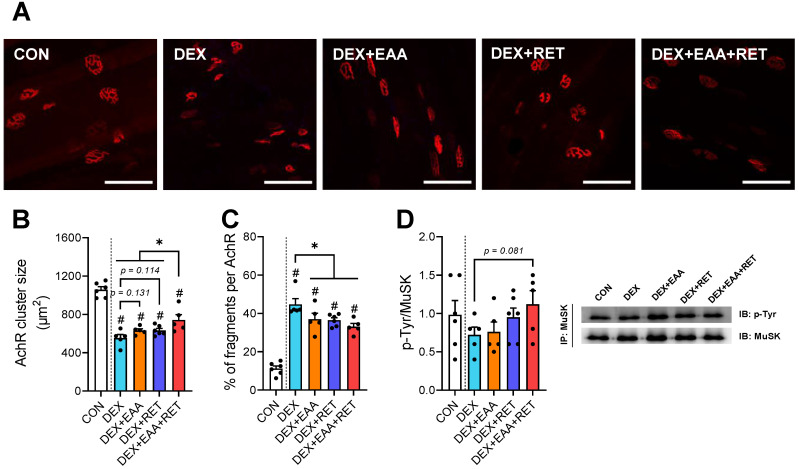
Combined treatment of EAA and RET restores a decrease in NMJ stability in skeletal muscle by DEX treatment. (**A**) Representative image of an acetylcholine receptor staining in plantaris. (n = 5–6). Scale bar, 100 μm. (**B**) Acetylcholine receptor cluster size in plantaris (n = 5–6). (**C**) Percentage of fragmented acetylcholine receptor in plantaris (n = 5–6). (**D**) Representative image and relative protein expression of Musk (n = 5–6). Data are presented as mean ± S.E. # Significant difference from control group (*p* < 0.05). * Significant difference between labeled groups (*p* < 0.05). CON, sedentary control; DEX, dexamethasone; EAAs, essential amino acids; RET, resistance exercise training; PLAN, plantaris muscle; AchR, acetylcholine receptor; MuSK, muscle-specific tyrosine kinase.

We show that combined treatment most effectively restores loss in muscle strength by DEX when compared to EAA or RET alone. Regarding this, we speculate that changes in the stability of NMJ and activation of the acetylcholine clustering pathway (MuSK pathway) might improve muscle strength by enhancing the transmission of nerve impulses from motor neurons to contracting muscles [11]. To investigate these possibilities, we analyzed the morphology of the acetylcholine receptor (AchR) and activity of MuSK signaling, a marker of NMJ stability [22]. In accordance with changes in muscle strength (Figure 1G,H), the DEX-induced declines in AchR cluster size tend to be restored by EAA (*p* = 0.138) or RET (*p* = 0.122), but synergistically and significantly by the combined treatment (Figure 3A,B). One of representative symptoms of myopathies is fragmentation of the AchR [23], which leads to muscle weakness [24]. Interestingly, we found that DEX increases the percentage of fragmented AchR, which is restored similarly by EAA, RET, or the combined treatment (Figure 3C). In addition, MuSK activation is attenuated by DEX, which is prevented partially by RET and fully by the combined treatment (vs. DEX, *p <* 0.108, vs. DEX, *p <* 0.089) (Figure 3D). Therefore, these results suggest that EAA and RET synergistically restore the loss of AchR cluster size and MuSK activity due to DEX treatment, which may contribute to improving muscle strength.

### 2.4. Combined Treatment of EAA and RET Prevents Muscle Fiber Type Shifting to Fast Glycolytic Fiber Due to DEX Treatment

**Figure 4:**
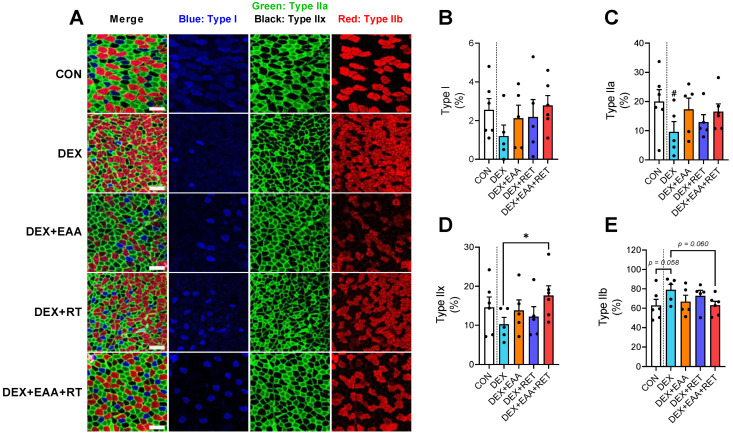
Combined treatment of EAA and RET prevents muscle fiber type shifting to fast glycolytic fiber due to DEX. (**A**) Representative image of immunofluorescence in gastrocnemius muscle for the triple-labeling with MHC type I (blue), MHC type IIa (green), MHC type IIx (black), and MHC type IIB (red). Scale bar, 100 μm (n = 5–6). (**B**) The proportion of MHC type-I-specific fibers (n = 5–6). (**C**) The proportion of MHC type-IIa-specific fibers (n = 5–6) (**D**) The proportion of MHC type-IIx-specific fibers (n = 5–6). (**E**) The proportion of MHC type-Iib-specific fibers (n = 5–6). Data are presented as mean ± S.E. # Significant difference from control group (*p* < 0.05). * Significant difference between labeled groups (*p* < 0.05). CON, sedentary control; DEX, dexamethasone; EAA, essential amino acids; RET, resistance exercise training; MHC, myosin heavy chain.

Muscle fiber types determine muscle contractility [25], and it is known that DEX treatment induces muscle fiber type transition [3]. Consistent with this, we found that DEX increases the proportion of fast glycolytic fibers (type IIb) while reducing the proportion of type I, IIa, and IIx, compared to control. These fiber type changes are prevented by the combined treatment (Figure 4A–E). Therefore, these data suggest that better maintenance of endurance capacity by the combined treatment may be due in part to better preservation of the muscle fiber type against shifting toward fast glycolytic fibers upon DEX treatment.

### 2.5. Combined Treatment of EAA and RET Completely Restores the Loss of Mitochondrial Biogenesis in Skeletal Muscle by DEX Treatment

**Figure 5:**
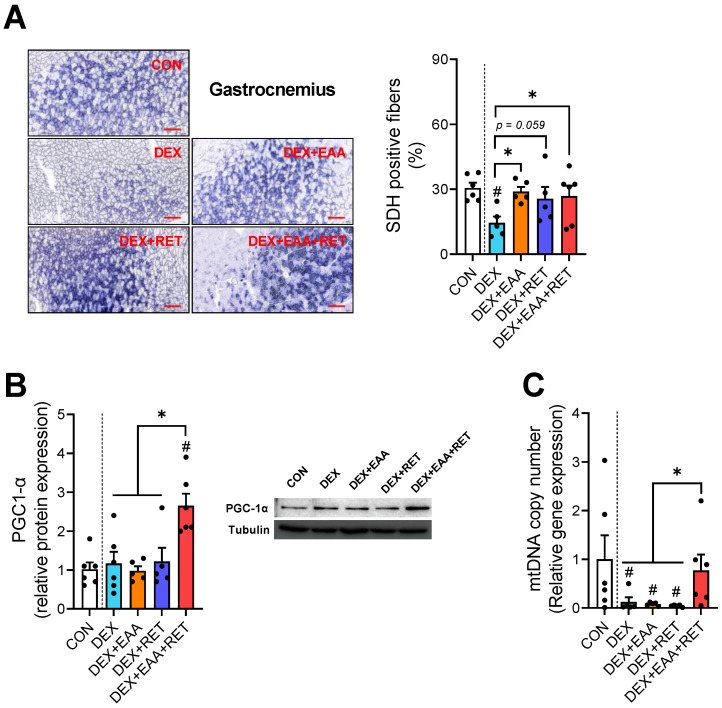
Combined treatment of EAA and RET completely restores the loss of mitochondrial biogenesis in skeletal muscle by DEX treatment. (**A**) Representative image and the proportion of SDH-positive muscle fibers in gastrocnemius (n = 5–6). Scale bar, 200 μm. (**B**) Representative image and relative protein expression of the PGC1-α in gastrocnemius (n = 5–6). (**C**) The relative mtDNA copy number with qPCR measurement in gastrocnemius (n = 5–6). Data are presented as mean ± S.E. # Significant difference from control group (*p* < 0.05). * Significant difference between labeled groups (*p* < 0.05). CON, sedentary control; DEX, dexamethasone; EAA, essential amino acids; RET, resistance exercise training; SDH, succinate dehydrogenase; PGC1-α, peroxisome proliferator-activated receptor gamma coactivator 1-alpha; mtDNA, mitochondrial DNA.

Mitochondrial biogenesis and oxidative capacity are important determinants for endurance capacity [17,26]. To test if improved endurance by EAA and/or RET is related to changes in mitochondrial biogenesis or oxidative capacity, we analyzed SDH enzyme activity and several markers of mitochondrial biogenesis. First, a succinate dehydrogenase (SDH) staining shows that DEX reduces SDH activity, however, it is completely restored by EAA, independent of the addition of RET (Figure 5A). Interestingly, we found that while DEX does not affect the expression of PGC1-α, the master regulator of mitochondrial biogenesis, the combined treatment of EAA and RET significantly increases the expression of PGC1-α protein (Figure 5B). Furthermore, the combined treatment completely prevents decreases in mitochondrial DNA content as the result of DEX treatment (Figure 5C). Therefore, the combined treatment of EAA and RET restores the loss of mitochondrial biogenesis in skeletal muscle due to DEX, which may lead to a full restoration of endurance capacity.

### 2.6. Combined Treatment of EAA and RET Completely Restores Impaired Insulin Sensitivity by DEX Treatment

**Figure 6:**
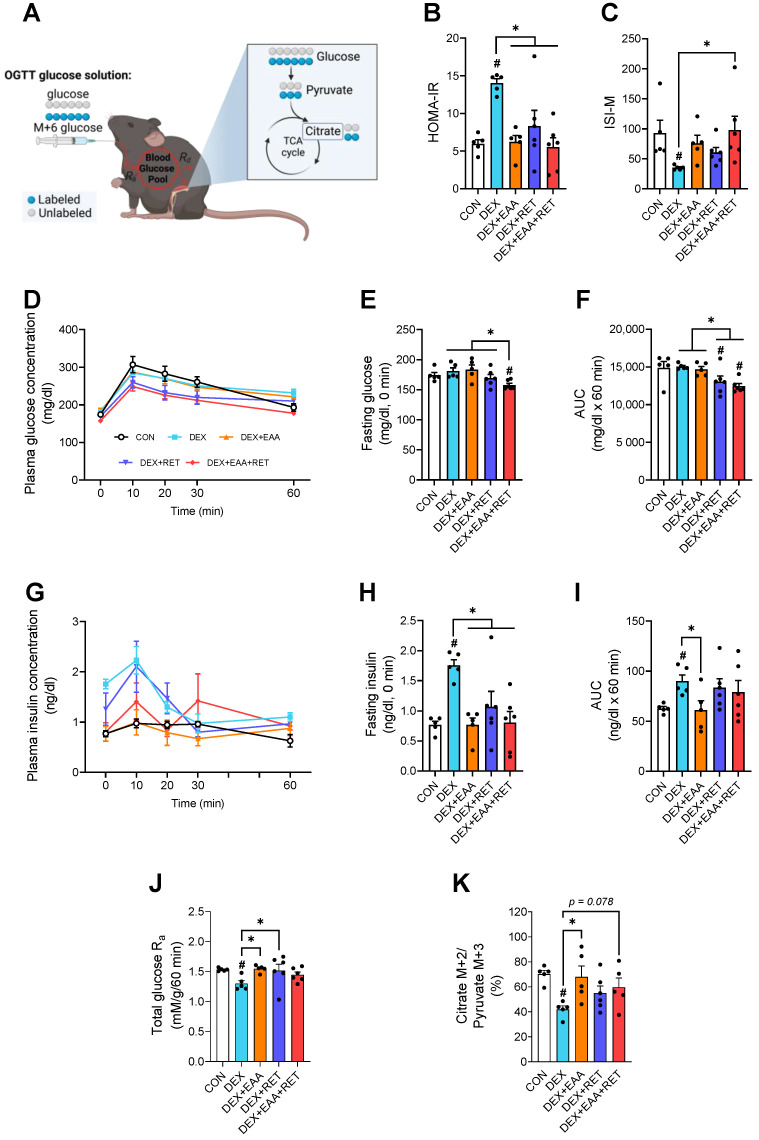
Combined treatment of EAA and RET completely restores impaired insulin sensitivity by DEX treatment. (**A**) Schematic showing glucose flux in vivo at systemic and myocellular levels using U-^13^C_6_ glucose tracer during OGTT. This figure was created with BioRender.com. (**B**) HOMA-IR index (n = 5–6). (**C**) Matsuda insulin sensitivity index (n = 5–6). (**D**) Plasma glucose concentration during the OGTT (n = 5–6). (**E**) Plasma glucose concentration at fasting state over 6 h (n = 5–6). (**F**) Area under the curve of plasma glucose concentration during OGTT (n = 5–6). (**G**) Plasma insulin concentration during the OGTT (n = 5–6). (**H**) Plasma insulin concentration at fasting state over 6 h (n = 5–6). (**I**) Area under the curve of plasma insulin concentration during OGTT (n = 5–6). (**J**) Total plasma glucose appearance during OGTT (n = 5–6). (**K**) Glucose flux into the Krebs cycle, reflected as the ratio of citrate M+2 to pyruvate M+3 in gastrocnemius muscle. Data are presented as mean ± S.E. # Significant difference from control group (*p* < 0.05). * Significant difference between labelled groups (*p* < 0.05). CON, sedentary control; DEX, dexamethasone; EAA, essential amino acids; RET, resistance exercise training; HOMA-IR, homeostasis model assessment of insulin resistance; ISI-M, Matsuda–DeFronzo insulin sensitivity index; AUC, area under the curve; glucose R_a_, rate of plasma glucose appearance.

Impaired glucose metabolism and insulin sensitivity are representative side effects of DEX treatment [27], and are attributed, in part, to a loss of muscle mass and/or quality, as skeletal muscle is responsible for the majority of postprandial glucose uptake [28]. Therefore, we hypothesized that the prevention of the DEX-induced declines in muscle mass and/or functions by EAA and/or RET would lead to a restoration of impaired glucose metabolism and insulin sensitivity. To test this hypothesis, we performed oral glucose tolerance tests (OGTT) with a simultaneous administration of a ^13^C_6_-labelled glucose tracer. Similar to our previous finding [4], we also found DEX-induced impairments of insulin resistance and sensitivity, assessed by HOMA-IR and Matsuda index, respectively (Figure 6B,C), which are associated with elevations in plasma concentrations of insulin, but not glucose, both at fasting and post-prandial states (Figure 6D–I). The impaired sensitivity or resistance of insulin is restored synergistically by EAA and RET (Figure 6D–I). Interestingly, it seems that the restoration of insulin sensitivity by the treatment of EAA and/or RET is differently regulated: the beneficial effect of EAA is accomplished through the suppression of plasma insulin concentrations, whereas that of RET is through the suppression of plasma glucose concentrations (Figure 6F,I).

To check the role of muscle mass changes in insulin sensitivity or resistance by respective treatment, the glucose metabolic flux in vivo was accessed using ^13^C_6_-labeled glucose during the OGTT. We first estimated the rate of total glucose appearance (*R_a_* glucose), that is summed the rates of endogenous and exogenous glucose appearance into the circulation, estimated by using an integral approach [29]. We found that DEX reduces *R_a_* glucose (Figure 6J), which is, however, reversed by EAA, RET, or the combined treatment. Given the fact that plasma glucose concentrations are not elevated by EAA and reduced by RET alone, or the combined treatment (Figure 6D–F), it is apparent that increases in rate of glucose disposal from the circulation to peripheral tissues such as skeletal muscle are increased to a greater extent with EAA, RET, or the combined treatment (Figure 6J). Consistent with this notion, we found that DEX decreases glucose flux into the Krebs cycle, assessed as the ratio of M+2 citrate to M+3 pyruvate, which is, however, reversed by EAA, RET, or the combined treatment (Figure 6K). Therefore, EAA, RET, or the combined EAA and RET treatment improve the impairment in glucose flux into the Krebs cycle due to DEX treatment, leading to the restoration of glucose metabolism to normal levels.

## 3. Discussion

Here we provide experimental evidence that dietary free EAA supplementation, particularly when combined with RET, can improve impaired MPS, mitochondrial biogenesis, NMJ stability, and fiber type preservation due to DEX treatment, leading to a full restoration of muscle strength and endurance, as well as whole-body insulin sensitivity. Thus, our findings suggest that free EAA supplementation, more potently when combined with RET, is useful for maintaining muscle mass and function in patients who routinely use DEX treatments.

The dysregulation of protein turnover dictates muscle atrophy (i.e., decreased protein synthesis relative to protein breakdown) [3,4]. As in our previous study [4], we found that DEX decreases muscle mass by ~20%, which is partially restored by EAA, RET, and, more robustly, the combined treatment. These improvements must be accomplished by changes in rates of MPS and/or MPB. In accordance with our previous study, in which EAA was provided in a context of mixed diet [4], free EAA supplementation also restores the DEX-induced decline in MPS, the effect becoming more robust when combined with RET in all types of muscle examined (i.e., GAS, SOL, and TA). Interestingly, we found that enhanced MPB, as reflected by protein ubiquitination in the muscle of DEX-treated mice, is inhibited only by EAA supplementation. Unlike ubiquitination, an increase in the expression of autophagy-related protein by DEX is inhibited by EAA and/or RET. Consistent with our findings, it is shown that 8 weeks of resistance exercise training suppresses autophagy without changing the UPS [30]. These results suggest that, given our previous finding from the EAA-mixed diet study [4], free EAA supplementation is a better way for protecting muscle against DEX treatment. Although we provide evidence of the better effects of free EAA supplementation, further studies are necessary not only to understand the difference in mechanisms underlying ways of consumption, but also to find the best consumption way to increase EAA effects. Taken together, it seems that the partial restoration of the DEX-induced loss in muscle mass is accomplished through different mechanisms, to some extent, which are regulated by EAA and RET: while EAA affects both MPS and MPB, RET affects MPS more. The differential regulation of the two main catabolic metabolisms (i.e., ubiquitin–proteasome and autophagy pathways) by RET need further investigations in the future.

Despite the similarly restored muscle mass among treatments, muscle strength is differentially improved among treatments, indicating that other factors are involved in the regulation of muscle quality. Previously, we reported that NMJ stability plays a critical role in muscle strength, independent of muscle mass [11]. Further, a study demonstrates that RET is sufficient to restructure the NMJ without muscle fiber hypertrophy or changes in fiber type [31]. Therefore, we speculated that the increase in muscle quality by RET and/or EAA is due, in part, to the structural improvement of AchR (i.e., NMJ stability) in an independent manner of muscle mass, leading to improving neural conduction from motor neuron to muscle fibers [32]. Consistent with the notion, we found that EAA and RET synergistically restore the DEX-induced decline in AchR cluster size, which is accompanied by a similar pattern on other markers of NMJ stability. Taken together, it seems that EAA and RET synergistically restore muscle strength via enhancing NMJ stability, despite the partial restoration of muscle mass.

In our previous study, the DEX-induced decline in endurance is not restored by EAA treatment when fed in a mixed diet [4]; surprisingly, we found in the present study that free EAA supplementation improves the loss in endurance capacity in DEX-treated mice. Furthermore, EAA and RET synergistically block the loss in mitochondrial number and function in skeletal muscle, leading to a full restoration of endurance capacity in DEX-treated mice. Interestingly, we found that protein expression of PGC1-α is increased only by the combined treatment in the muscle of DEX-treated mice. Since over-expression of PGC1-α shifts fiber types toward more oxidative fibers and enhanced muscle oxidative metabolism and endurance capacity [33,34,35], an increase in PGC1-α expression by the combined treatments appears to be involved in muscle function and endurance capacity. Taken together, the combined treatment of EAA and RET blocks DEX-induced fiber type conversion and loss of mitochondrial biogenesis in skeletal muscle, leading to improving endurance capacity in the mice.

In our previous study, we show that loss in muscle mass induced by DEX treatment worsens insulin resistance and glucose homeostasis [4]. In accordance with this, we found in the present study that EAA or RET is sufficient to restore impaired insulin sensitivity, with more robust effects with the combined treatment. However, free EAA supplementation, dissimilar to an EAA-enriched diet, has no glucose-attenuating effect but instead results in a complete restoration of insulin concentrations to normal levels in both fasted and post-prandial states (i.e., during OGTT). While it is not clear how these divergent results occur, depending upon the ways of EAA feeding (i.e., free EAA form vs. EAAs in mixed diet), it is possible that free EAA form vs. EAAs in mixed diet may provide a more favorable condition (i.e., a reduced insulin response) for facilitating gluconeogenesis from amino acids, particularly alanine, which is shown to increase in DEX treatment [36]. Interestingly, we found that glucose concentrations during the OGTT are decreased in RET or the combined treatment, or at least not increased in EAA alone despite increased *R_a_* glucose, which is the summed rates of appearance of both exogenous (from the OGTT solution) and endogenous glucose (largely from hepatic glucose output). This indicates that, regardless of the exact underlying mechanisms, the improved insulin sensitivity in EAA and/or RET might result in a greater peripheral glucose uptake and/or subsequent oxidation by tissues, particularly skeletal muscle, reflected as a greater labelling (enrichment) of citrate relative to pyruvate in muscle. Therefore, it is likely that an increase in mitochondrial biogenesis, along with an increase in muscle mass, improves glucose metabolism and insulin sensitivity.

In the present study, we show that free EAA supplementation and RET synergistically block the DEX-induced declines in muscle strength, endurance, and insulin sensitivity. Therefore, we suggest that EAA supplementation is useful for maintaining muscle mass and function in patients who routinely use DEX treatment. However, there are several limitations in the current study, including potential differences regarding timing of treatment [37] and sex difference [38]. Furthermore, it is important to confirm our findings in clinical outcome trials.

## 4. Materials and Methods

### 4.1. Animal Care and Experiment Design

We randomly assigned the C57BL/6J male mice at the age of 9 weeks to five groups of sedentary control (CON, N = 5–6), dexamethasone (DEX, N = 5–6), dexamethasone treat + essential amino acids (DEX+EAA, N = 5–6), dexamethasone treat + resistance exercise training (DEX+RET, N = 5–6), and dexamethasone treat +EAA with RET (DEX+EAA+RET, N = 5–6). EAA was orally administered twice a day (1.5 g/kg/day) for 2 weeks. The detailed EAA composition is presented Table 1.

### 4.2. Resistance Exercise Training Protocol

Resistance exercise was performed through ladder climbing [11]. Briefly, mice climbed the ladder at 50%, 70%, 90%, and 100% of weight of their previous exercise session’s weight, followed by up to 10 reps, increasing the weight by 3 g with each session. In case of failure, exercise was performed with the previous weight. Between repetitions, the mice rested for 2 min.

### 4.3. Physical Function Tests

Maximum carrying capacity (MCC) testing was performed before and after the intervention, following three consecutive days of familiarization. During the MCC test, the mouse climbed the ladder at 50%, 70%, 90%, and 100% of the body weight, and after that, a load of 3 g was applied until the mouse could not climb the ladder. Between repetitions, the mouse rested for 2 min at the top of the ladder.

Grip strength tests were measured using a grip strength meter (DEFII-002, Chatillon, Largo, FL, USA) before and after the intervention. Briefly, after a mice grasped a wire grid with their four limbs, the tail was gently pulled back until it was completely detached from the grid. The measurement was repeated 5 times, and the maximum force was reflected in the results.

To evaluate the endurance ability, a familiarization session (10 m/min 10 min, 13 m/min 5 min, 17 m/min 3 min, 10% incline) was conducted for 3 consecutive days before and after the intervention, and then an exhaustion test was performed using a motorized treadmill (1050-RM Exer-3/6, Columbus Instruments, Columbus, OH, USA). The mice started running at a speed of 8 m/min at a slope of 10%, and the speed was increased to 1 m/min every 2 min until exhaustion [39]. Criterion of exhaustion was defined as the time at which the mouse stayed for more than 5 seconds after falling to the electric grid without attempting to run despite the physical stimulus.

### 4.4. Immunoblot Analysis

Immunoblotting was performed as previously described [11]. Briefly, muscles were denatured in lysis buffer and protein concentrations were measured using a BCA assay kit (#A53225, Thermo Fisher Scientific, Waltham, MA, USA). A total of 25 µg of protein lysate was separated by sodium dodecyl sulfate polyacrylamide gel electrophoresis (SDS-PAGE), and transferred to a polyvinylidene fluoride (PVDF) membrane. Primary antibodies, including mTOR (1:1000, #2983), p-mTOR (Ser2448) (1:1000, #2971), rps6 (1:1000, #9272), p-rps6 (Ser473) (1:1000, #9271), ubiquitin (1:1000, #3933S), ATG7 (1:1000, #8558, Cell signaling technology, MA, USA), LC3B (1:1000, #NB100-2220, Novus Biologicals, CO, USA), PGC1-α (1:1000, ab54481, abcam, Cambridge, UK), MuSK (1:1000, sc-134398), p-Tyr (1:1000, sc-508, Santa Cruz Biotechnology, Santa Cruz, CA, USA), and β-tubulin (1:5000, #05-661, Merck Millipore, Burlington, MA, USA), were incubated overnight at 4 °C. Then, the membrane was washed with TBS-T and incubated with a secondary antibody at room temperature for 1 h. After immunodetection using ECL detect reagent (GE healthcare, Buckingham, UK), protein was quantified using ImageJ image analysis software (National Institutes of Health).

### 4.5. Immunofluorescence Staining

To measure myofiber CSA or fiber type composition, muscle sections were incubated overnight at 4 ℃ with rabbit anti-laminin (1:500, #L9393, Sigma-Aldrich, St Louis, MO, USA), MHC type I (1:50, #BA-F8), MHC type IIa (1:500, #SC-71), and MHC type IIb (1:100, #BF-FC, DSHB, Iowa City, Iowa, USA). After washing in PBS, sections were incubated with Alexa Fluor 405-conjugated anti-rabbit IgG (1:500, #Z25213), Alexa Fluor 488-conjugated anti-rabbit IgG (1:500, #A-21121), Alexa Fluor 555-conjugated anti-rabbit IgG (1:500, #A-21426, Invitrogen, Waltham, MA, USA) for 1 h. Images were acquired using a confocal microscope (Zeiss LSM 700, Zeiss, Oberkochen, Germany) in the same setting. Image J (National Institutes of Health) image analysis software was used for fiber CSA calculations. 

### 4.6. Immunohistochemistry

Mitochondrial oxidative capacity was measured with succinate dehydrogenase (SDH) staining. Briefly, muscle sections were incubated for 1 hour at 37 °C with working solution (0.2 M phosphate buffer, 0.2 M sodium succinate buffer, and tetranitro blue tetrazolium).

### 4.7. Mitochondrial DNA (mtDNA) Copy Number Quantification

DNA was extracted from gastrocnemius muscle sample using QIAamp DNA minikit (Qiagen, Hilden, Germany), in accordance with manufacturer’s instructions, and quantified by Nanodrop2000 spectrometer (Thermoscientific, Wilmington, NC, USA). Then, 25 ng total DNA was amplified with TOPrealTM qPCR premix (SYBR Green with high ROX) (Enzynimics, Daejeon, Republic of Korea), The primers sequence used for the mtDNA(mtMDA4), and internal control (28S rRNA) were as follows: mtNDA4 forward primer, CTCCTCAGTTAGCCACATAGCA, and reverse primer, TGTGGATCCGTTCGTAGTTGGA, 28s rRNA forward primer, AGGACCCGAAAGATGGTGAACTA, and reverse primer, CGGAGGGAACCAGCTACTAGAT. mtDNA copy number was determined using 2^−ΔΔCt^ method.

### 4.8. Isolation of Myofibrillar Protein

To isolate myofibrils, a differential centrifugation method was used, as previously described [40]. Briefly, each muscle (soleus, gastrocnemius, and tibialis anterior) was homogenized in a buffer (550 mM KCI, 5 mM EGTA, and 100 mM MOPS) and centrifuged at 800× *g* for 5 min at 4 °C to precipitate the myofibril fraction. The separated supernatant was used for further analysis.

### 4.9. D_2_O Labeling and Calculations of Protein Synthetic Rate

All mice were administered an i.p. bolus injection of 35 µL/g of 99% D_2_O (DLM-4, Cambridge isotope laboratories, Tewsbury, MA, USA) with 0.9% NaCL, as previously described [41], followed by free access to drinking water enriched to 8% D_2_O for two weeks. Calculation of muscle protein fractional synthesis rate (FSR, %/time) was predicated on the precursor–product relation [42].

FSR (%/t) = [(E_Ala_)/(EBW × 3.7)] × 100 (1)

k_s_ = −ln (1 − FSR)/t (2)

Absolute synthesis (mg/day × 14 days) = k_s_ (day − 1) × muscle pool size (mg) × 14 days (3)

where enrichment (E) is expressed as mole percent excess (MPE) calculated as a tracer to tracee ratio (TTR)/(TTR + 1); t is time of labeling; and ks represents the fractional synthesis rate constant [43]. The absolute synthesis rate of muscle protein was calculated by multiplying the cumulative ks over 14 days of the labeling period by muscle protein pool size with the assumption of 12% of total muscle wet weight, which is myofibrillar protein in muscle [44].

### 4.10. Stable Isotope Enrichment Analysis

Enrichment of stable isotopes in body fluids and muscles was analyzed, as previously described [45,46]. Briefly, the ^2^H enrichment in plasma was measured using the acetone exchange method, and for alanine enrichment analysis, the tissues were homogenized with 6% perchloric acid, precipitated at 21,000× *g* at 4 °C, and hydrolyzed to amino acids at 100 °C for 16 h. The hydrolyzed free amino acids were extracted using a cation-exchange resin column and dried using Speed vac (Savant Instruments, Farmingdale, NY, USA). In addition, to measure the glycolysis pathway and TCA cycle metabolic flux in muscle, frozen gastrocnemius muscle immediately after dissection was homogenized with 70% ACN and 30% distilled water, and centrifuged at 14,000× *g* at 4 °C for 5 min. Centrifuged supernatant was transferred to a new tube and dried using a Speed vac. Derivatization by GC-MS was started in 50 µL of 2% MOX in pyridine for 90 min at 37 °C, 50 µL of MTBSFTA + 1% TBDMSCIS was added and incubated at 60 °C for 30 min. After that, the sample was centrifuged at 14,000× *g* for 5 min at room temperature, and the supernatant was transferred to a GC-MS vial.

### 4.11. Oral Glucose Tolerance Test and Whole-Body Glucose Metabolism Calculation

To measure glucose metabolism, mice were orally administered with [U-^13^C_6_]glucose tracer (1.5 g/kg, Cambridge Isotope Laboratories, Andover, MA, USA) following 6 h fasting. Blood was collected from the tail tip at a time prior to −120 min, and 10, 20, 30, and 60 min after the oral glucose tracer administration. Plasma glucose concentration was immediately measured using a glucometer (GM9, Analox Instrument, Stoubridge, UK). Plasma insulin concentration was measured using an insulin ELISA kit, in accordance with manufacturer’s instructions (80-INSMSU-E10, ALPCO, Salem, MA, USA). Total rate of glucose appearance (*R_a_*) was calculated based on the single pool kinetic using an integral approach [29].

(4) $$Ra=\frac{Dose}{\int_{0}^{60}E\left(t\right)d\left(t\right)}$$

where 60 is the end time point of MPE during glucose tolerance, 0 is the baseline value over MPE, and dose is the amount of glucose in the oral gavage. Insulin resistance was calculated using homeostasis model assessment of insulin resistance (HOMA-IR), and Matsuda insulin sensitivity index was used for an index of insulin sensitivity [47].

### 4.12. Statistical Analysis

One-way ANOVA was performed to validate DEX effects among all groups and main effects among four DEX groups on variables, and Fisher’s least significant difference (LSD) post hoc test was used for multiple comparisons. Data were expressed as mean ± S.E. and analyzed using GraphPad Prism 9 and SPSS for window version 21.0. The area under the curve (AUC) for blood glucose concentration, enrichment, and insulin concentration during OGTT was determined using the trapezoidal method. Statistical significance was set at *p* < 0.05.

## 5. Conclusions

In conclusion, our data suggest that the feeding of free, balanced, essential amino acids and resistance exercise training can synergistically restore the DEX-induced declines in muscle strength and endurance, in part through improvements of myofibrillar protein synthesis, mitochondrial biogenesis, neuromuscular junction stability, and muscle fiber type preservation, all of which may lead to the full restoration of whole-body insulin sensitivity. These findings suggest the therapeutic potential of EAA and/or RET for a variety of clinical conditions where DEX is used as a part of medical treatment.

## Figures and Tables

**Table 1:** Composition of essential amino acids.

Amino Acid	Percentage (%)	Dietary Intake (g/kg/day)
Histidine	10	0.150
Isoleucine	10	0.150
Leucine	21	0.315
Lysine	18	0.270
Methionine	4	0.060
Phenylalanine	12	0.180
Threonine	14	0.210
Valine	10	0.150
Tryptophan	1	0.015
Total	100	1.5

## Data Availability

The datasets used and/or analyzed during in this study are available from the corresponding author upon reasonable request.
